# LncRNA *AC021683.2* promotes chemotherapy resistance in acute myeloid leukemia

**DOI:** 10.1016/j.isci.2025.113439

**Published:** 2025-08-26

**Authors:** Lan Li, Xue-Ni An, Yong-Tong Ruan, Xin Liang, Jing-Lan Hao, Guang-Xun Gao, Jun-Fang Bi, Yan-Rong Gao, Xiao Lu, Hai-Long Tang, Ping Gao, Xiao-Ming Dong

**Affiliations:** 1 College of Life Sciences, Shaanxi Normal University, Xi’an 710119, China; 2 Department of Hematology, Xijing Hospital, Fourth Military Medical University, Xi’an 710032, China; 3 Department of Combined Traditional Chinese Medicine and West Medicine, Shijiazhuang Traditional Chinese Medicine Hospital, Shijiazhuang 050051, China; 4 Shaanxi Rehabilitation Hospital, Xi’an 710065, China; 5 Key Laboratory of the Ministry of Education for Medicinal Resources and Natural Pharmaceutical Chemistry, Shaanxi Normal University, Xi’an 710119, China

**Keywords:** Pharmacology, Biochemistry, Cancer

## Abstract

Acute myeloid leukemia (AML) is an aggressive clonal malignancy of hematopoietic progenitors with poor clinical outcomes. Though many patients respond well to induction chemotherapy, relapse occurs. The mechanisms underlying AML chemoresistance remain unclear. In our study, we performed whole transcriptome sequencing (WTS) on diagnosed AML samples sensitive or resistant to IA (idarubicin and cytarabine) induction treatment. We observed that lncRNA *AC021683.2* is upregulated in IA-resistant patients and associated with poor prognosis. *AC021683.2* depletion increased the chemosensitivity of AML cells to Ara-C both *in vitro* and *in vivo* by accelerating BCLAF1 ubiquitination and degradation, and *AC021683.2* depletion enhanced sensitivity partially by depending on BCLAF1. Both *AC021683.2* and BCLAF1 positively correlated with RAD50, which mediated their roles in Ara-C-resistant AML cells. These findings demonstrated that the lncRNA *AC021683.2* enhances the resistance of AML/Ara-C-resistant cells to Ara-C *in vitro* and *in vivo*, offering a potential target for treating Ara-C-resistant AML.

## Highlights

• LncRNA *AC021683.2* is elevated in patients with IA-resistant AML
• LncRNA *AC021683.2* depletion enhances Ara-C sensitivity *in vitro/vivo*
• LncRNA *AC021683.2* knockdown accelerates ubiquitination and degradation of BCLAF1
• RAD50 mediates LncRNA *AC021683.2*/BCLAF1-driven chemoresistance in AML

## Graphical abstract

**Figure:**
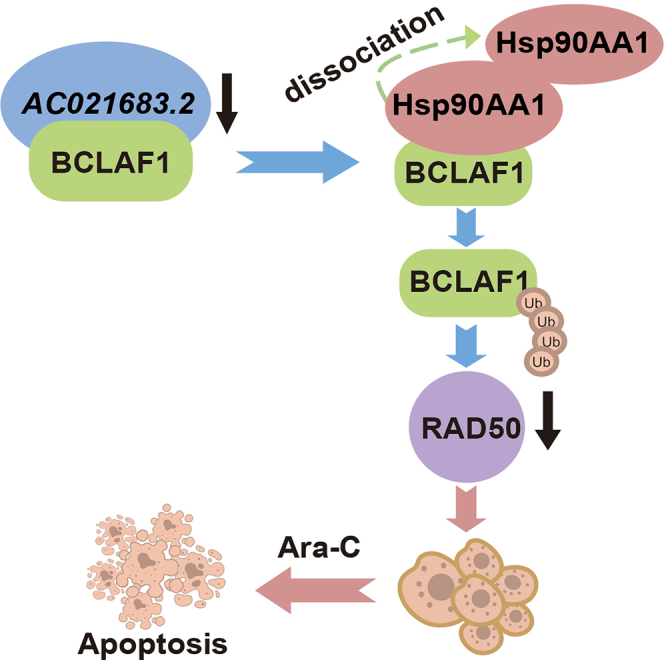

## Introduction

Acute myeloid leukemia (AML) is a group of heterogeneous hematologic malignancies, characterized by abnormal clonal expansion and undifferentiated myeloid precursor cells.^1^ At present, combination chemotherapy, such as cytarabine, idarubine, and doxorubicin, is still the main treatment method for AML,^2^^,^^3^ however, nearly half of the initial chemotherapy responders will eventually relapse,^4^^,^^5^ which challenges the chemotherapy in clinical treatment effect.

Cytarabine, also known as Ara-C, interferes with DNA synthesis, causes stalling of replication forks, and DNA strand breaks, triggering the DNA damage response mechanisms of cells.^6^ The mechanisms of DNA damage response include activation of cell cycle checkpoints, DNA repair and apoptosis.^7^ Currently, Ara-C is a major component of primary and salvage chemotherapy regimens for AML.^8^^,^^9^ Although cytarabine has demonstrated clinical benefit in diagnosis AML patients, chemotherapy resistance and toxicity are still the major cause for poor prognosis in AML patients.^10^^,^^11^ Therefore, it is necessary to identify key targets/pathways and combination therapy strategies to overcome drug resistance and disease recurrence in AML.

Long non-coding RNAs (lncRNAs) are considered as potentially therapeutic, diagnostic, and prognostic factors in AML.^12^ Dysregulation of lncRNAs expression can affect various downstream targets that are responsible for AML proliferation, differentiation, and drug resistance.^13^^,^^14^ It was reported that lncRNAs play critical roles in the development and induction of the chemoresistance in patients with AML.^15^ However, the biological roles of most lncRNAs in chemotherapy resistance of AML are not fully understood. Thus, exploring the long non-coding RNA and the regulatory mechanisms in chemo resistance process will provide potentially useful therapeutic targets for treatment of drug resistance in patients with AML.

Increasing evidence indicates that BCL-2-associated transcription factor 1 (BCLAF1) exerts pleiotropic regulatory functions across diverse biological processes, including pre-mRNA splicing,^16^ mRNA maturation,^17^ DNA damage response,^16^^,^^18^ and gene transcription.^19^ For example, in hepatocellular carcinoma pathogenesis, BCLAF1 promotes tumor progression by stabilizing *C-MYC* mRNA^20^ and transcriptionally activating HIF-1α,^21^ thereby orchestrating oncogenic signaling networks. Lee et al. demonstrated that BCLAF1 regulates apoptosis and DNA repair through γH2AX-mediated pathways.^18^ Additionally, The LNCRNA *LNCCIRBIL* fights coronary artery disease reperfusion injury by binding to BCLAF1 and inhibiting its nuclear translocation.^22^ Notably, while these findings highlight BCLAF1’s multifaceted roles in cellular homeostasis and disease, its specific contribution to Ara-C response in AML remains undefined.

In this study, we first collected and isolated mononuclear cells from the bone marrow of diagnosed AML patients that were sensitive (*n* = 5) or resistant (*n* = 5) to IA induction treatment, respectively. Then, we performed the whole transcriptome sequencing and found that a lncRNA *AC021683.2* is upregulated in patients with IA-resistant AML. *AC021683.2* depletion augments chemosensitivity of AML cells to Ara-C *in vitro* and *in vivo*. Mechanistically, *AC021683.2* interacts with and regulates BCLAF1 expression in the presence of Ara-C. In addition, *AC021683.2* depletion enhances the chemosensitivity of HL60-ADR cells to Ara-C partially by depending on BCLAF1. Finally, we found that DNA repair protein RAD50 mediates the role of *AC021683.2* and BCLAF1 in Ara-C-induced HL60-ADR cells apoptosis. Our findings provided evidence that *AC021683.2* functions as a lncRNA by regulating BCLAF1 in AML, and revealed that targeting the *AC021683.2*/BACLAF1/RAD50 axis provides potential strategy for the treatment of resistance to Ara-C in AML.

## Results

### The expression of *AC021683.2* is elevated in patients with IA-resistant AML

**Figure 1:**
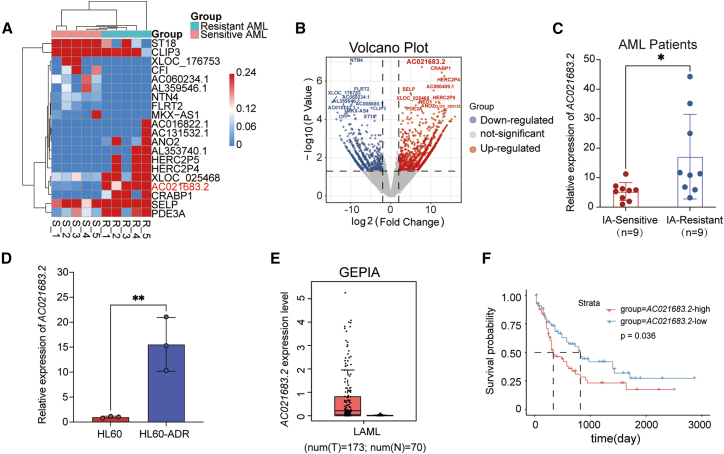
The expression of *AC021683.2* is elevated in patients with IA-resistant AML Sensitive and resistant samples to IA induction treatment from diagnosed AML patients were collected for whole transcriptome sequencing. (A) Hierarchical clustering heatmap depicting individual samples and gene expression differences between IA-sensitive and IA-resistant samples (*n* = 5 per group). (B) A volcano plot of differentially expressed genes between IA-sensitive and IA-resistant samples (|log2 fold change|> 2, adjusted *p* value (Padj) < 0.05). (C) qRT-PCR analysis of expression levels of *AC021683.2* in diagnosed AML patients that were sensitive (*n* = 9) or resistant (*n* = 9) to IA induction treatment. (D) qRT-PCR analysis of expression levels of *AC021683.2* in the HL60 and HL60-ADR cells. (E) *AC021683.2* expression in AML and normal group from GEPIA database. (F) Association between survival of AML patients and *AC021683.2* levels from the online UCSC Xena database. *p* values were assessed using Gehan-Breslow-Wilcoxon test. Data are represented as means ± S.D. ∗*p* < 0.05; ∗∗*p* < 0.01. *p* values were assessed using two-tailed Student’s *t* tests (C–D).

To determine the mechanisms of chemotherapy resistance in AML, we performed whole transcriptome sequencing from diagnosed AML samples (Table 1) that were sensitive or resistant to IA induction treatment. The expression of 43218 genes above detectable expression levels (>1 read in at least one sample) was compared in a pairwise approach between IA-sensitive (*n* = 5) and IA-resistant (*n* = 5) samples. This resulted in 2787 differentially expressed genes (*p* ≤ 0.05) (Figures 1A and 1B) (Table S1). As *AC021683.2* was top one ranked gene, we further validated the expression patterns of *AC021683.2* in an additional IA-resistant (*n* = 9) and IA-sensitive (*n* = 9) patient samples (Table S2). Consistent with transcriptome sequencing data, we confirmed that *AC021683.2* is highly expressed in IA-resistant samples (*n* = 9) compared with IA-sensitive samples (*n* = 9) (Figure 1C). Then, we also observed that *AC021683.2* was more abundant in Ara-C-resistant HL60-ADR cells compared to HL60 cells (Figure 1D). Furthermore, we analyzed the expression levels of *AC021683.2* in normal and AML samples from the GEPIA dataset, the data showed that the expression of *AC021683.2* was elevated in AML patients (Figure 1E). Moreover, the highly expressed *AC021683.2* was correlated with the overall survival of AML patients in the strata cohort (Figure 1F). These results suggested that lncRNA *AC021683.2* could play an important role in chemotherapy resistance in AML.

**Table 1:** Characteristics of AML samples analyzed by whole transcriptome sequencing

Characteristics	No. of AML
Number of patients	10
Age (median, years)	40 (18–58)

**Gender**	

Male	3
Female	7

**WHO diagnosis**	

Acute myelomonocytic leukemia	2
AML with maturation	4
AML with CBFB::MYH11	2
Acute megakaryoblastic leukemia	1
AML with RUNX1:RUNX1T1	1

**Status**	

Sensitive	5
Resistant	5

**Source**	

Bone marrow (BM)	10

**Blast percentage (median)**	

Sensitive	74.24
Resistant	72.88

**Karyotype**	

MLL/(AF17/AF1Q/AF1PAFXSEPT6)	1
Normal	4
CBFB/MYH11	1
Inv (16), +22	1
MLL/AF17	1
6q+, 7q-, −10, 11q-	1
AML1/ETO	1

**Consolidation therapy**	

IA	10

### *AC021683.2* depletion augments chemosensitivity of AML to Ara-C *in vitro* and *in vivo*

**Figure 2:**
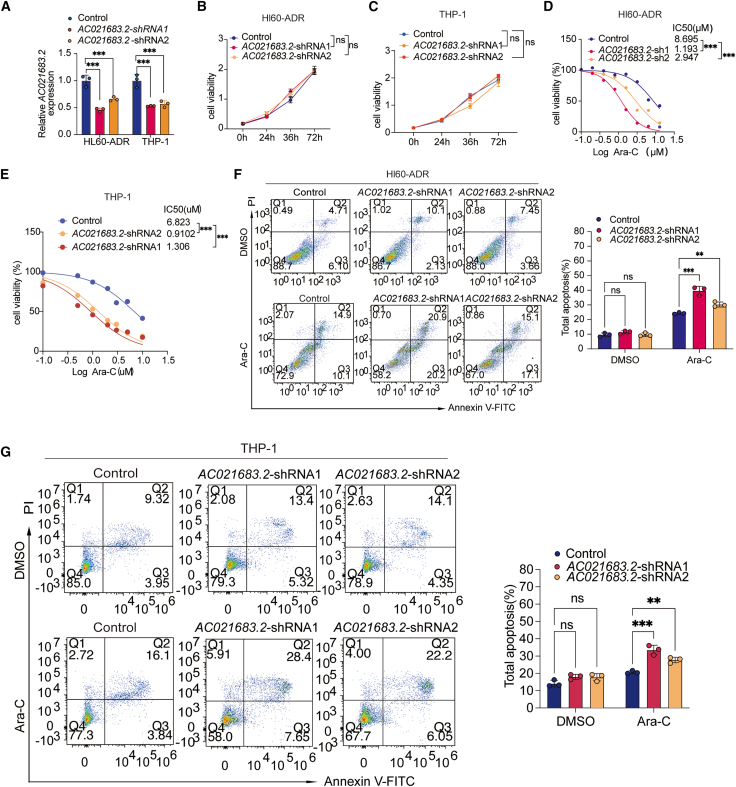
*AC021683.2* depletion augments chemosensitivity of AML cell lines to Ara-C *in vitro* (A) RT-qPCR analysis of *AC021683.2* mRNA levels in control and *AC021683.2* knockdown HL60-ADR cells and THP-1 cells. (B) Knockdown of *AC021683.2* had no effect on the cell viability measured by CCK-8 assays (mean ± SD of triplicate experiments) in HL60-ADR cells. (C) Knockdown of *AC021683.2* had no effect on the cell viability measured by CCK-8 assays (mean ± SD of triplicate experiments) in THP-1 cells. (D) *AC021683.2* depleted HL60-ADR cells or control cells were treated with different concentrations of Ara-C for 48 h, the cell viability was detected by CCK-8 assay and the IC50 was calculated. (E) *AC021683.2* depleted THP-1 cells or control cells were treated with different concentrations of Ara-C for 48 h, the cell viability was detected by CCK-8 assay and the IC50 was calculated. (F) *AC021683.2* inhibited HL60-ADR cells or control cells were treated with Ara-C (5 μM) for 48 h, the cell apoptosis was detected by flow cytometry. (G) *AC021683.2* knockdown THP-1 cells or control cells were treated with Ara-C (5 μM) for 48 h, the cell apoptosis was measured by flow cytometry. Data are represented as mean ± S.D. from triplicate experiments. ∗*p* < 0.05; ∗∗*p* < 0.01; ∗∗∗*p* < 0.001. *p* values were assessed using two-way ANOVA (A, D-G) and two-tailed Student’s *t* tests (B-C).

To investigate *AC021683.2*’s role in AML chemoresistance, we established stable *AC021683.2* knockdown in Ara-C-resistant HL60-ADR and THP-1 cells using lentiviral shRNA. RT-qPCR assay confirmed that the *AC021683.2* shRNA lentivirus in these two AML cell lines significantly decreased the endogenous *AC021683.2* levels (Figure 2A). CCK8 assay indicated that *AC021683.2* knockdown didn’t affect the cell viability of HL60-ADR and THP-1 (Figures 2B and 2C). However, *AC021683.2* knockdown markedly enhanced cellular sensitivity in HL60-ADR and THP-1 cells under treatment with Ara-C (Figures 2D and 2E). Furthermore, cell apoptosis was evaluated using propidium iodide (PI)/Annexin V staining. In HL60-ADR cells, *AC021683.2* depletion did not affect cell apoptosis in the absence of Ara-C (Figure 2F). Following 2 days of Ara-C treatment, the total apoptosis rate was 25% in control shRNA cells, which increased to 41.1% in *AC021683.2* shRNA1 cells and 32.2% in *AC021683.2* shRNA2 cells, suggesting that knockdown of *AC021683.2* increased the sensitivity of HL60-ADR cells to Ara-C (Figure 2F). Moreover, PI/Annexin V staining in THP-1 cells also confirmed that the inhibition of *AC021683.2* increased Ara-C-induced apoptosis (Figure 2G).

**Figure 3:**
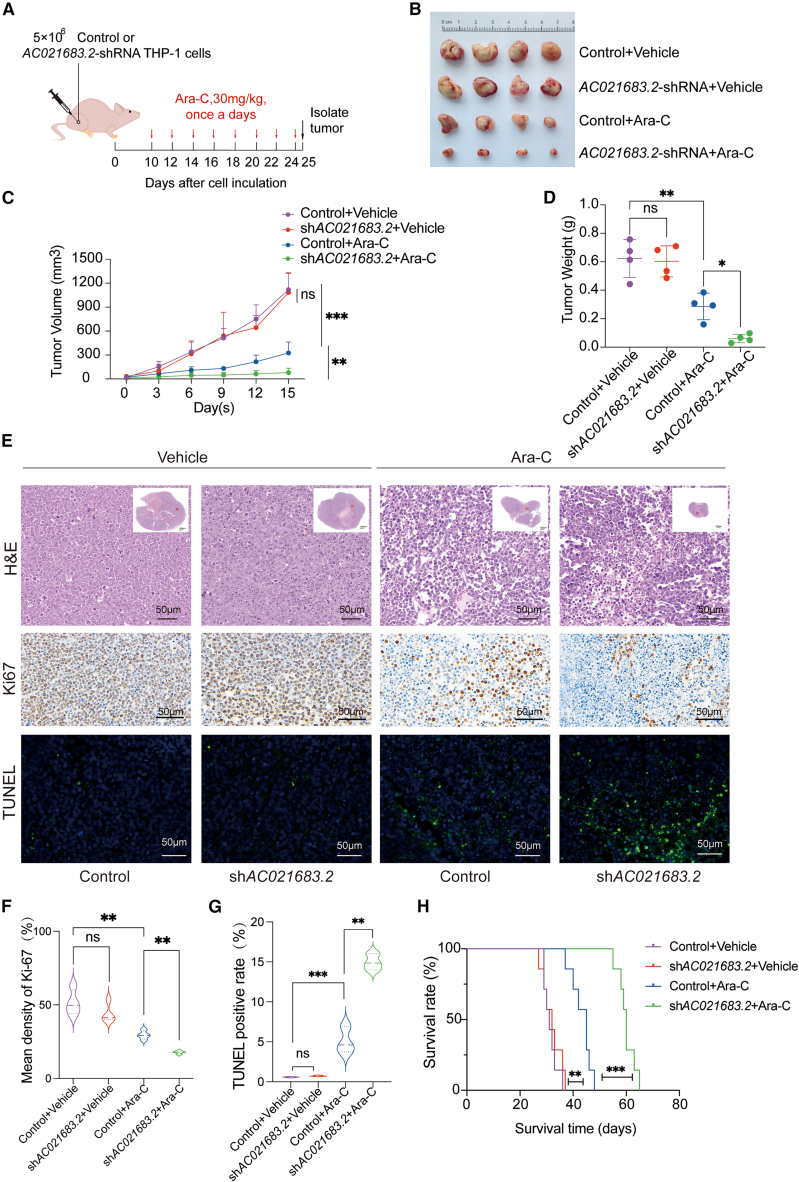
*AC021683.2* suppression enhances chemosensitivity of AML cells to Ara-C *in vivo* (A) Schematic of subcutaneous tumorigenesis. 5×10^6^ THP-1 cells stably expressing sh*AC021683.2* or control shRNA were injected into the flank of nude mice. The mice were divided into four groups: control, sh*AC02683.2*, control + Ara-C, sh*AC021683.2* + Ara-C (*n* = 4 in each group). When tumors were visible, mice were injected intraperitoneally with Arc-C or Vehicle (50 mg/kg/day). (B) Images from subcutaneous xenografts of mice in the control, sh*AC021683.2*, control + Ara-C, and sh*AC021683.2* + Ara-C groups. (C) Tumor volume growth curve of subcutaneous xenografts. (D) Tumor weight of subcutaneous xenografts. (E) The representative H&E, Ki-67 and TUNEL staining images of tumor sections from Control, sh*AC021683.2*, Control + Ara-C, and sh*AC021683.2* + Ara-C groups, scale bar = 50 μm. (F and G) The statistical quantification of IHC staining of Ki-67 (F) and TUNEL positive rate (G) in each group. (H) Survival curve of mice in the control, sh*AC021683.2*, control + Ara-C, and sh*AC021683.2* + Ara-C groups (*n* = 7 in each group). Kaplan-Meier survival analyses were performed with the log-rank test. Data are represented as mean ± S.D. from triplicate experiments. ∗*p* < 0.05; ∗∗*p* < 0.01; ∗∗∗*p* < 0.001. *p* values were assessed using two-way ANOVA (C) and two-tailed Student’s *t* tests (D, F, and G).

To confirm the effect of *AC021683.2* on AML cells to Ara-C *in vivo,* BALB/c-nude mouse xenograft models were established by subcutaneous injection of sh*AC021683.2* or control shRNA THP-1 cells. Successful tumorigenic mice received treatment with Ara-C (Figure 3A). As shown in Figure 3B, knockdown of *AC021683.2* significantly inhibited tumor growth compared to the control group with Ara-C treatment. Meanwhile, suppression of *AC021683.2* effectively reduced tumor volume (Figure 3C) and tumor weight (Figure 3D) (*p* < 0.05). Next, hematoxylin and eosin (H&E) staining was applied to evaluate the histomorphological changes within the tumor tissues. The results revealed that *AC021683.2* suppression markedly induced cell apoptosis under Ara-C treatment (Figure 3E). Furthermore, xenografts derived from the sh*AC021683.2* group exhibited a lower expression of Ki-67 and a higher proportion of TUNEL-positive cells compared to the control group under Ara-C treatment (Figures 3E–3G). Moreover, we assessed the therapeutic efficacy of Ara-C by injecting mice via the tail vein with THP-1 cells stably expressing either sh*AC021683.2* or control shRNA. The results demonstrated no significant difference in mean survival time between the control and sh*AC021683.2* groups in the absence of Ara-C treatment. However, mice with *AC021683.2* knockdown exhibited a significantly prolonged survival compared to controls following Ara-C treatment (Figure 3H), indicating that *AC021683.2* knockdown enhances the therapeutic efficacy of Ara-C. Collectively, the depletion of *AC021683.2* augments the sensitivity of AML to Ara-C both *in vitro* and *in vivo*.

### *AC021683.2* knockdown accelerates ubiquitination and degradation of BCLAF1 in HL60-ADR cells under treatment with Ara-C

**Figure 4:**
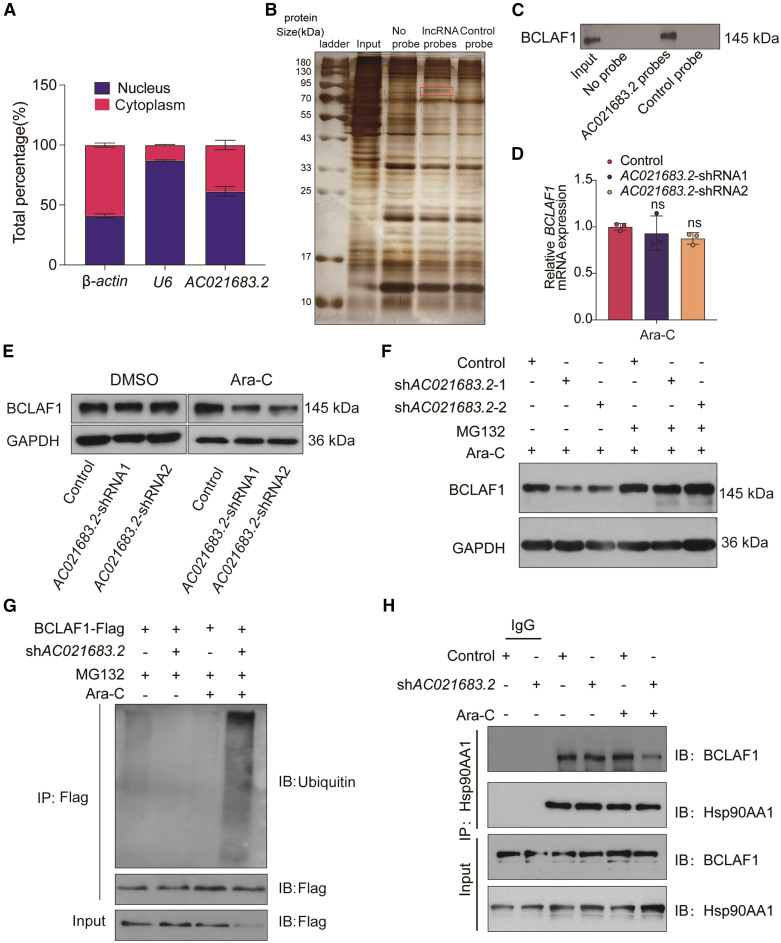
*AC021683.2* knockdown accelerates ubiquitination and degradation of BCLAF1 in HL60-ADR cells under treatment with Ara-C (A) RT-qPCR was used to detect the subcellular fractions of *AC021683.2* in HL60-ADR cells. *U6* and *β-actin* were used as markers of the nucleus and cytoplasm, respectively. (B) Silver staining of *AC021683.2* RNA pull-down proteins. A probe non-specific for *AC021683.2* was used as control. Mass spectrometry analysis identified BCLAF1 (red arrow) as a protein that interacts with *AC021683.2*. (C) *AC021683.2* RNA pull-down followed by western blot validated the interaction with BCLAF1. (D) RT-qPCR analysis of *BCLAF1* mRNA levels in control and *AC021683.2* knockdown HL60-ADR cells in the presence of 5 μM Ara-C for 48 h (Data are represented as mean ± S.D. from triplicate experiments). (E) Western blotting analysis of BCLAF1 protein levels in control or *AC021683.2*-knockdown HL60-ADR cells treated with or without (5 μM) Ara-C for 48 h. GAPDH was used as the internal control. (F) Western blotting analysis of BCLAF1 expression levels in *AC021683.2* knockdown or control HL60-ADR cells treated with or without MG132 (20 μM) for 8 h in the presence of 5 μM Ara-C. GAPDH was used as the internal control. (G) The ubiquitination of BCLAF1 was analyzed by immunoprecipitation in *AC021683.2*-knockdown Flag-BCLAF1 HL60 cells treated with or without (5 μM) Ara-C and western blot with indicated antibodies. Cell lysates were subjected to immunoblot with FLAG tag antibody. (H) *AC021683.2* depleted or control HL60-ADR cells were treated with or without (5 μM) Ara-C for 48 h, and immunoprecipitations were performed with indicated antibodies. ns, no significance.

To further investigate the functional mechanism of *AC021683.2* in chemotherapy resistance in AML, we first determined the localization of *AC021683.2* in HL60-ADR cells. RT-qPCR results showed that *AC021683.2* is a whole cell localized RNA (Figure 4A). Then, we performed native RNA pull-down assay using biotinylated DNA probes against *AC021683.2*. Silver staining of the pull-down protein complexes identified one unique protein bands at ∼80 kDa compared with the control pull-down. Mass spectrometry analysis identified the unique protein band as BCLAF1 (Figure 4B). We then validated this interaction by western blotting for BCLAF1 after *AC021683.2* RNA pull-down, confirming a direct interaction between *AC021683.2* and BCLAF1 (Figure 4C). We then evaluated the effect of *AC021683.2* on BCLAF1 expression levels. RT-qPCR assay suggested that *AC021683.2* knockdown did not alter *BCLAF1* mRNA level (Figure 4D), whereas western blot assay showed that knockdown of *AC021683.2* reduces BCLAF1 protein levels in HL60-ADR cells treated with Ara-C (Figure 4E). Besides, in Ara-C-treated HL60-ADR cells, MG132 (a ubiquitin-proteasome inhibitor) blocked BCLAF1 degradation caused by *AC021683.2* inhibition (Figure 4F), indicating *AC021683.2* stabilizes BCLAF1 via proteasome pathway. To confirm the findings, we knocked down *AC021683.2* in BCLAF1-Flag-HL60 cells and measured endogenous BCLAF1 ubiquitination. Results showed that *AC021683.2* knockdown in Ara-C-treated HL60-ADR cells increased BCLAF1 ubiquitination (Figure 4G). Hsp90AA1 was previously shown to protect BCLAF1 from degradation,^20^ so we tested whether *AC021683.2* disrupts their interaction. Immunoprecipitation revealed that *AC021683.2* knockdown in Ara-C-treated HL60-ADR cells weakened Hsp90AA1-BCLAF1 binding (Figure 4H). Taken together, *AC021683.2* knockdown promotes BCLAF1 ubiquitination and degradation in HL60-ADR cells under treatment with Ara-C.

### BCLAF1 suppresses chemosensitivity of HL60 cells to Ara-C

**Figure 5:**
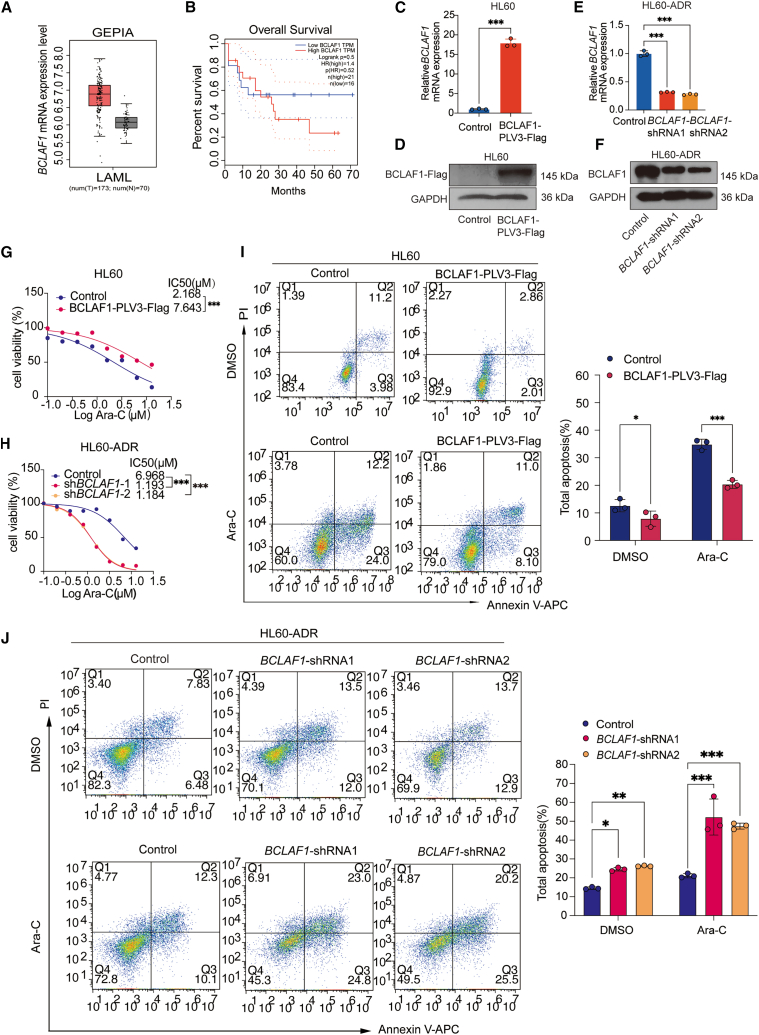
BCLAF1 suppresses chemosensitivity of HL60-ADR cells to Ara-C (A) BCLAF1 expression in AML and normal group from GEPIA database. (B) Association between survival of AML patients and BCLAF1 expression levels from the TCGA database. *p* values were assessed using Gehan-Breslow-Wilcoxon test. (C) RT-qPCR analysis of mRNA levels of *BCLAF1* in control and *BCLAF1-* overexpressing HL60 cells. (D) Western blotting analysis of BCLAF1 protein levels in control and BCLAF1-overexpressing HL60 cells. (E) RT-qPCR analysis of mRNA levels of *BCLAF1* in control and *BCLAF1* inhibited HL60-ADR cells. (F) Western blotting analysis of BCLAF1 protein levels in control and BCLAF1 knockdown HL60-ADR cells. (G) BCLAF1 overexpressed HL60 cells or control cells were treated with different concentrations of Ara-C for 48 h, the cell viability was detected by CCK-8 assay and the IC50 was calculated. (H) BCLAF1 depleted HL60-ADR cells or control cells were treated with different concentrations of Ara-C for 48 h, the cell viability was detected by CCK-8 assay and the IC50 was calculated. (I) BCLAF1 overexpressed HL60 cells or control cells were treated with or without Ara-C (5 μM) for 48 h, the cell apoptosis was detected by flow cytometry. (J) BCLAF1 knockdown HL60-ADR cells or control cells were treated with or without (5 μM) Ara-C for 48 h, the cell apoptosis was measured by flow cytometry. Data are represented as mean ± S.D. from triplicate experiments. ∗*p* < 0.05; ∗∗*p* < 0.01; ∗∗∗*p* < 0.001. *p* values were assessed using two-tailed Student’s *t* tests (C, E) and two-way ANOVA (G-J).

To evaluate the functional role of BCLAF1 in AML, we examined BCLAF1 expression levels in normal and AML samples from GEPIA dataset. We observed that BCLAF1 is upregulated in patients with AML compared with normal group (Figure 5A). The highly expressed BCLAF1 was found to be correlated with the decreased overall survival of AML patients in the TCGA cohort (Figure 5B), indicating that the prognosis for patients with highly expressed BCLAF1 is poor.

To further study the role of BCLAF1 in chemotherapy resistance in AML, we established cell lines with BCLAF1 overexpression and knockdown in HL60 and HL60-ADR cells. RT-qPCR and western blot analysis demonstrated that the BCLAF1-PLV3 lentivirus effectively increased *BCLAF1* mRNA and protein levels (Figures 5C and 5D), while the shRNA lentivirus endogenous BCLAF1 mRNA and protein levels (Figures 5E and 5F). CCK8 assay indicated that BCLAF1 overexpression enhanced the viability of HL60 cells under treatment with Ara-C (Figure 5G), whereas knockdown of BCLAF1 inhibited Ara-C-treated HL60-ADR cells viability (Figure 5H). Furthermore, propidium iodide (PI) ⁄Annexin V staining was performed to detect the effect of BCLAF1 on HL60 cell apoptosis. In the absence of Ara-C, control cells exhibited an apoptosis rate of 15.18%, compared to only 4.87% in cells overexpressing BCLAF1; When Ara-C was present, the apoptosis rates increased to 36.2% in control cells versus 19.1% in BCLAF1-overexpressing cells, suggesting that BCLAF1 overexpression decreased the sensitivity of HL60 cells to Ara-C (Figure 5I). In HL60-ADR cells without Ara-C treatment, knockdown of BCLAF1 resulted in an increase in apoptosis from 14.31% to 25.5% (*BCLAF1* shRNA1) and 26.6% (*BCLAF1* shRNA2). Following 2 days of Ara-C treatment, the total apoptosis rate was 22.4% in control shRNA cells, which increased to 47.8% in *AC021683.2* shRNA1 cells and 45.7% in *AC021683.2* shRNA2 cells (Figure 5J), suggesting that BCLAF1 knockdown increased the sensitivity of HL60-ADR cells to Ara-C. Altogether, the highly expressed BCLAF1 was correlated with poor prognosis for AML patients and decreased the sensitivity of HL60 cells to Ara-C.

### *AC021683.2* depletion enhances the chemosensitivity of HL60-ADR cells to Ara-C partially by depending on BCLAF1

**Figure 6:**
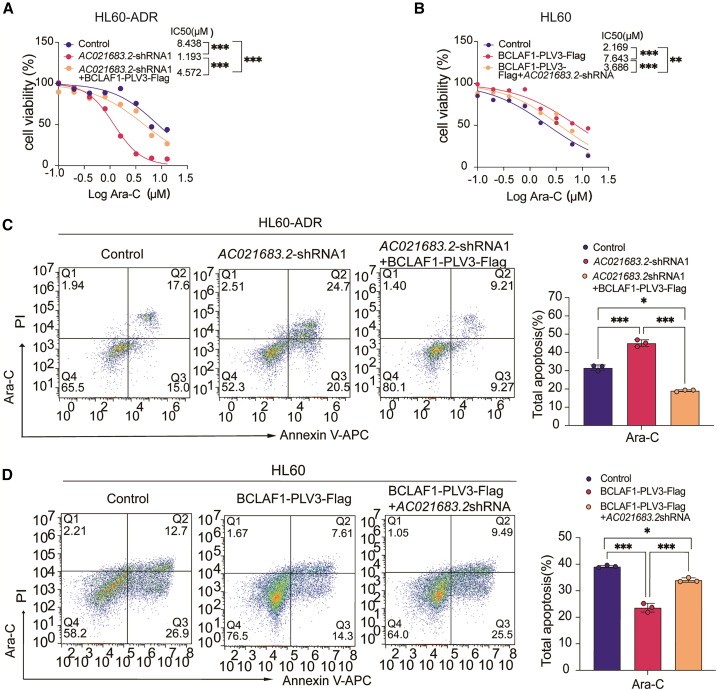
*AC021683.2* depletion enhances the chemosensitivity of HL60-ADR cells to Ara-C partially by depending on BCLAF1 (A) The cell viability was detected by CCK8 assay in *AC021683.2* knockdown HL60-ADR cells with BCLAF1 overexpression stimulated with different concentrations of Ara-C for 48 h. (B) The cell viability was detected by CCK8 assay in BCLAF1 overexpressed HL60 cells with *AC021683.2* knockdown stimulated with different concentrations of Ara-C for 48 h. (C) The indicated cell lines (control, *AC021683.2* shRNA, *AC021683.2* shRNA with BCLAF1 overexpression) were stimulated with (5 μM) Ara-C for 48 h, the cell apoptosis was measured by flow cytometry. (D) The indicated cell lines (control, BCLAF1 overexpression, BCLAF1 overexpression with *AC021683.2* shRNA) were treated with (5 μM) Ara-C for 48 h, the cell apoptosis was measured by flow cytometry. Data are represented as mean ± S.D. from triplicate experiments. ∗*p* < 0.05; ∗∗*p* < 0.01; ∗∗∗*p* < 0.001. *p* values were assessed using two-way ANOVA (A–D).

We further examined whether there is a direct relationship between BCLAF1 and Ara-C-induced apoptosis in *AC021683.2* knockdown HL60-ADR cells. Then, we re-expressed BCLAF1 in *AC021683.2*-knockdown HL60-ADR cells by infecting BCLAF1 overexpression lentivirus. As expected, re-expression of BCLAF1 partially rescued the increased sensitivity of *AC021683.2* knockdown HL60-ADR cells to Ara-C (Figure 6A). We also inhibited *AC021683.2* expression in BCLAF1 overexpression HL60 cells by infecting *AC021683.2* shRNA lentivirus. As expected, *AC021683.2* inhibition partially rescued the decreased sensitivity of BCLAF1 overexpressed HL60 cells to Ara-C (Figure 6B). Furthermore, propidium iodide (PI) ⁄Annexin V staining was performed to detect the apoptosis of the indicated HL60-ADR cell strains. The total apoptosis rate observed in Ara-C-treated HL60-ADR cells infected with control shRNA lentivirus was 32.6%, which increased to 45.2% in HL60-ADR cells with *AC021683.2* knockdown. The re-expression of BCLAF1 in *AC021683.2*-knockdown cells mitigated Ara-C-induced apoptosis, resulting in a decrease of the apoptosis rate to 18.48% (Figure 6C). Similarly, in HL60 cells treated with Ara-C, the group infected with control lentivirus exhibited a total apoptosis rate of 39.6%, while overexpression of BCLAF1 reduced this rate to 21.91%. Notably, the suppression of *AC021683.2* in HL60 cells overexpressing BCLAF1 led to an increase in Ara-C-induced apoptosis, elevating the rate to 34.99% (Figure 6D). The results confirmed that *AC021683.2* knockdown enhances the chemosensitivity of AML cells to Ara-C partially by depending on BCLAF1.

### *AC021683.2* or BCLAF1 is positively correlated with RAD50 in AML

**Figure 7:**
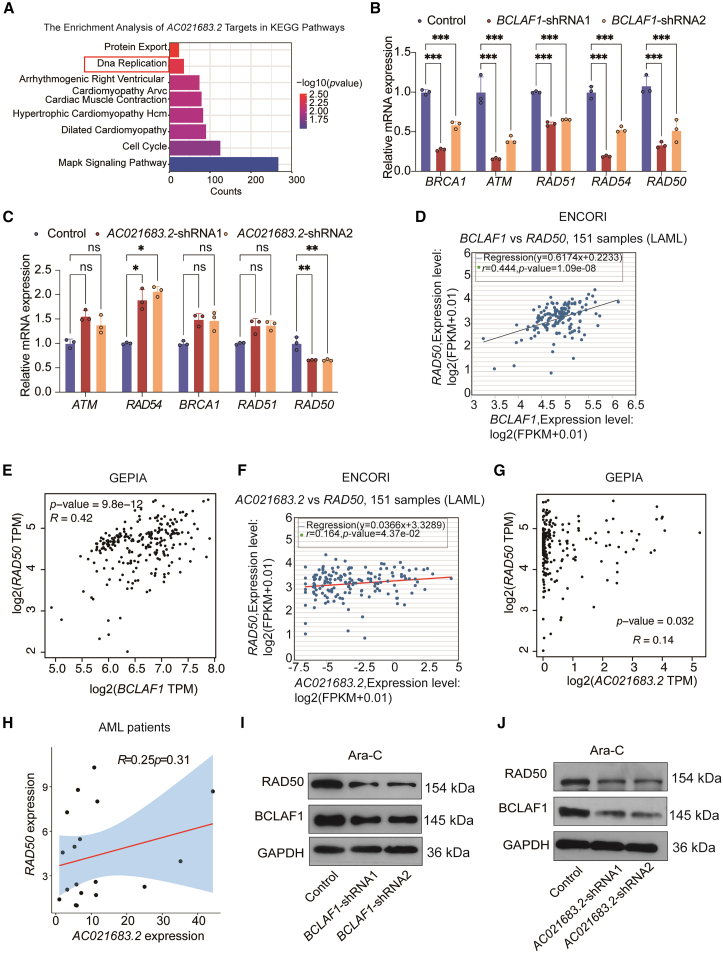
*AC021683.2* or BCLAF1 is positively correlated with RAD50 in AML (A) The KEGG pathway enrichment analysis for the targets of the identified *AC021683.2* from ENCORI database. (B) RT-qPCR analysis of mRNA levels of *BRCA1*, *ATM*, *RAD51*, *RAD54*, and *RAD50* in control and BCLAF1 suppressed HL60-ADR cells in the presence of 5 μM Ara-C. (C) RT-qPCR analysis of mRNA levels of *BRCA1*, *ATM*, *RAD51*, *RAD54*, and *RAD50* in control and *AC021683.2* inhibited HL60-ADR cells in the presence of 5 μM Ara-C. (D) Co-expression analysis of *RAD50* and *BCLAF1* was performed in AML samples based on the ENCORI database. (E) Co-expression analysis of *RAD50* and *BCLAF1* was performed in AML samples based on the GEPIA database. (F) Co-expression analysis of *AC021683.2* and *RAD50* in AML samples was performed based on the ENCORI database. (G) Co-expression analysis of *AC021683.2* and *RAD50* in AML samples was performed based on the GEPIA database. (H) Pearson correlations between *AC021683.2* and *RAD50* expression levels in AML patient samples. (I) Western blotting analysis of BCLAF1 and RAD50 protein levels in control and BCLAF1 knockdown HL60-ADR cells in the presence of 5 μM Ara-C, respectively. (J) Western blotting analysis of BCLAF1 and RAD50 protein levels in AC02683.2 suppressed HL60-ADR cells and control cells under stimulation with 5 μM Ara-C. Data are represented as mean ± S.D. from triplicate experiments. ∗*p* < 0.05; ∗∗*p* < 0.01; ∗∗∗*p* < 0.001. *p* values were assessed using two-way ANOVA (B–C). ns, no significance.

As Ara-C interferes with DNA synthesis and DNA strand breaks, triggering the DNA damage response mechanism of cells.^6^ We performed KEGG pathway enrichment analysis on the targets of *AC021683.2* from ENCORI. The results indicated notable enrichment in the protein export and DNA replication pathways (Figure 7A), suggesting a connection between *AC021683.2*/BCLAF1-mediated chemotherapy resistance in AML and DNA damage repair. RT-qPCR analysis of DNA repair-related genes revealed that BCLAF1 knockdown markedly suppressed the mRNA levels of *BRCA1*, *ATM*, *RAD51*, *RAD54*, and *RAD50* in HL60-ADR cells treated with 5 μM Ara-C (Figure 7B). In contrast, *AC021683.2* knockdown only resulted in a reduction of RAD50 expression (Figure 7C). Correlation analyses conducted using the ENCORI and GEPIA databases demonstrated positive associations between *BCLAF1* and *RAD50* (Figures 7D and 7E), as well as between *AC021683.2* and *RAD50* in AML samples (Figures 7F and 7G), which were validated in IA-resistant (*n* = 9) and IA-sensitive (*n* = 9) patient samples (Figure 7H). Furthermore, Western blotting confirmed that BCLAF1 inhibition led to a decrease in RAD50 protein levels (Figure 7I), while *AC021683.2* knockdown concurrently reduced the expression levels of both BCLAF1 and RAD50 (Figure 7J). Collectively, these results indicated that *AC021683.2* might promote the resistance of AML cells to Ara-C through RAD50.

### RAD50 mediates the role of *AC021683.2* and BCLAF1 in ara-C-induced HL60-ADR cells apoptosis

**Figure 8:**
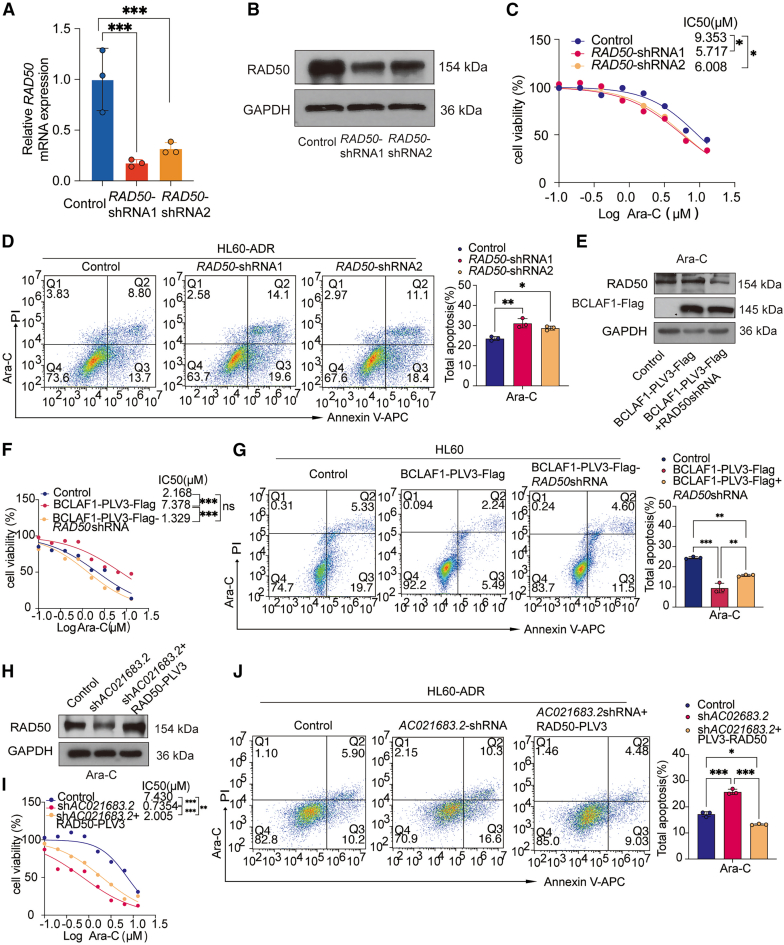
RAD50 mediates the role of *AC021683.2* and BCLAF1 in Ara-C-induced HL60-ADR cells apoptosis (A) RT-qPCR analysis of mRNA levels of *RAD50* in control and *RAD50* suppressed HL60-ADR cells. (B) Western blotting analysis of RAD50 protein levels in HL60-ADR cells with RAD50 knockdown and control cells. (C) RAD50 depleted HL60-ADR cells or control cells were treated with different concentrations of Ara-C for 48 h, respectively, the cell viability was detected by CCK-8 assay and the IC50 was calculated. (D) RAD50 knockdown HL60-ADR cells or control cells were treated with (5 μM) Ara-C for 48 h, respectively, the cell apoptosis was measured by Flow cytometry. (E) Western blotting analysis of RAD50 protein levels and BCLAF1-Flag in BCLAF1 overexpressed HL60 cells with RAD50 knockdown and control cells. (F) The cell viability was detected by CCK8 assay in BCLAF1 overexpressed HL60 cells with RAD50 knockdown stimulated with different concentrations of Ara-C for 48 h. (G) The indicated cell lines (Control, BCLAF1 overexpression, BCLAF1 overexpression with *RAD50* shRNA) were treated with (5 μM) Ara-C for 48 h, respectively, the cell apoptosis was measured by flow cytometry. (H) Western blotting analysis of RAD50 protein levels in *AC021683.2* knocked down HL60-ADR cells with RAD50 overexpression and control cells. (I) The cell viability was detected by CCK8 assay in *AC021683.2* knocked down HL60-ADR cells with RAD50 overexpression stimulated with different concentrations of Ara-C for 48 h. (J) The indicated cell lines (Control, *AC021683.2* shRNA, *AC021683.2* shRNA with RAD50 overexpression) were stimulated with (5 μM) Ara-C for 48 h, respectively, the cell apoptosis was measured by Flow cytometry. Data are represented as mean ± S.D. from triplicate experiments. ∗*p* < 0.05; ∗∗*p* < 0.01; ∗∗∗*p* < 0.001. *p* values were assessed using two-tailed Student’s *t* tests (A) and two-way ANOVA (C-D, F-G, and I-J). ns, no significance.

To characterize the role of RAD50 in *AC021683.2*/BCLAF1-mediated Ara-C resistance, *RAD50* shRNA lentivirus was used to knockdown RAD50 expression in HL60-ADR cells. Western blot and qRT-PCR confirmed notable reduction of endogenous RAD50 protein and mRNA levels (Figures 8A and 8B). CCK8 assays showed that RAD50 knockdown inhibited viability of Ara-C-treated HL60-ADR cells (Figure 8C), while PI/Annexin V staining revealed increased apoptosis (Figure 8D), indicating that RAD50 inhibition enhanced chemosensitivity to Ara-C. In BCLAF1-overexpressing HL60 cells, RAD50 knockdown reversed the protective effect of BCLAF1 on Ara-C sensitivity. Western blot validated RAD50 depletion and BCLAF1-Flag expression in treated cells (Figure 8E), while CCK8 assays showed that knockdown of RAD50 reversed the weakened chemosensitivity of HL60 cells to Ara-C caused by BCLAF1 overexpression (Figure 8F). Apoptosis analysis confirmed that RAD50 deletion in BCLAF1-overexpressing cells rescued Ara-C-induced apoptosis, with total apoptosis increasing from 7.73% to 16.1% (Figure 8G). Moreover, western blot verified RAD50 overexpression in treated cells (Figure 8H), and CCK8 assays showed that RAD50 overexpression in *AC021683.2*-knockdown HL60-ADR cells partially reversed the enhanced chemosensitivity to Ara-C (Figure 8I). Apoptosis staining revealed that RAD50 re-expression reduced Ara-C-induced apoptosis from 26.9% to 13.51% in *AC021683.2*-knockdown cells (Figure 8J). Collectively, RAD50 mediated the role of *AC021683.2* and BCLAF1 in Ara-C-induced HL60-ADR cells apoptosis.

## Discussion

In the present study, we performed whole-transcriptome sequencing of diagnosed AML samples with sensitivity or resistance to IA (idarubicin + cytarabine) induction treatment to identify more non-coding RNA in chemotherapy resistance. We found that lncRNA *AC021683.2* expression was significantly higher in IA-resistant samples (*n* = 5) than in IA-sensitive samples (*n* = 5). Similarly, when compared with IA-sensitive AML patients (*n* = 9), IA-resistant AML patients (*n* = 9) showed higher transcriptional levels of *AC021683.2*, a trend also observed in drug-resistant cells. Furthermore, knockdown of *AC021683.2* enhanced Ara-C sensitivity in Ara-C-resistant AML cells, which was consistent with our sequencing data. Notably, elevated *AC021683.2* expression was associated with poor prognosis in AML patients.

LncRNAs are involved in regulating multiple biological processes, including proliferation, apoptosis, DNA damage repair and chemotherapeutic resistance.^23^^,^^24^^,^^25^ For example, the lncRNA *MACC1-AS1* promotes fatty acid oxidation-dependent GC stem cell properties and chemotherapy resistance by negatively regulating *miR-145-5p*, inhibiting reactive oxygen species production and apoptosis of GC cells.^26^ The lncRNA *SNHG29* inhibits the development of FLT3-ITD AML by modulating EP300-associated histone acetylation modification.^27^ In our study, we found that knockdown of *AC021683.2* did not affect AML cells proliferation, but enhanced Ara-C sensitivity by inducing apoptosis. *In vivo* experiments showed that AML-bearing mice with *AC021683.2* knockdown exhibited increased sensitivity to Ara-C. The results suggested that *AC021683.2* depletion augments chemosensitivity of AML cells to Ara-C.

The regulatory mechanisms of lncRNAs encompass both transcriptional and post-transcriptional regulatory networks, orchestrating gene expression through molecular interactions and structural rearrangements.^28^ At the transcriptional level, *cis*-acting lncRNAs influence gene expression via elements such as enhancers and RNA-DNA triplex structures, whereas *trans*-acting lncRNAs recruit transcription factors or epigenetic modifier complexes to alter chromatin states or transcription factor activity.^29^^,^^30^ In post-transcriptional regulation, nuclear lncRNAs participate in mRNA processing and splicing, while cytoplasmic lncRNAs modulate translational efficiency by binding to ribosomes, sequestering microRNAs (miRNAs), or regulating protein functions.^31^^,^^32^ For example, *HOXA-AS2* can directly bind to EZH2, inhibiting proliferation and inducing differentiation in AML by modulating LATS2 expression.^33^ Furthermore, lncRNA *CD27-AS1* influences AML cell viability via the *miR-224-5p*/PBX3/MAPK signaling pathway.^34^ To uncover how high *AC021683.2* expression boosts Ara-C resistance in AML cells, we used RNA pull-down assays to identify its binding partner, BCLAF1. The interaction between *AC021683.2* and BCLAF1 was also verified. Our results also revealed that *AC021683.2* inhibition accelerated the ubiquitination and degradation of BCLAF1 in the presence of Ara-C, which was halted by MG132. These results suggested that *AC021683.2* might control the expression of BCLAF1 by regulating post-translational modifications of BCLAF1.

BCLAF1 plays a key role in regulating biological processes such as apoptosis and DNA damage repair.^18^^,^^35^ A previous study showed that mir-194-5p binds to the 3′-UTR of *BCLAF1* to inhibit BCLAF1 expression and regulate cell cycle progression and apoptosis in AML cells.^36^ BCLAF1 also induces cisplatin resistance in lung cancer cells by regulating DNA damage repair^37^ In our study, we found that high BCLAF1 expression correlates with poor AML prognosis and enhances Ara-C resistance. Notably, BCLAF1 overexpression rescued the sensitivity induced by *AC021683.2* knockdown, thereby expanding its role in chemoresistance.

RAD50 has been shown to be involved in the regulation of DNA damage repair and is associated with tumor prognosis.^38^^,^^39^ Targeting RAD50 is a chemotherapy-sensitive approach to cancer therapy in the context of chemotherapy resistance.^40^ In our study, the depletion of *AC021683.2* or BCLAF1 resulted in reduced *RAD50* mRNA and protein levels in AML cells treated with Ara-C. RAD50 knockdown enhanced HL60-ADR sensitivity to Ara-C, while its overexpression rescued the increased sensitivity caused by *AC021683.2* depletion. Additionally, RAD50 knockdown partially reversed the resistance induced by BCLAF1 overexpression. Our results suggested that *AC021683.2* enhanced the resistance of AML/Ara-C-resistant cells to Ara-C by regulating the expression of RAD50. However, how *AC021683.2* or BCLAF1 regulates RAD50 expression needs further investigation.

For over four decades, Ara-C has remained the cornerstone of intensive therapy for AML, with high-dose cytarabine (HiDAC) protocols—typically administered at 1–3 g/m^2^ every 12 h for 3–6 days. Administered at 10–20 times standard induction doses, HiDAC boosts intracellular ara-CTP levels to counteract tumor cell drug efflux and metabolic inactivation.^41^^,^^42^^,^^43^ However, clinical trials show that dose escalation beyond 3 g/m^2^ fails to improve overall survival,^44^ likely because drug-resistant leukemic cells activate DNA damage repair pathways or evade apoptosis, rendering HiDAC ineffective while exacerbating myelosuppression and multi-organ toxicity.^45^ Age is a key determinant of HiDAC tolerability: in patients ≥60 years, declining organ function and elevated baseline inflammation worsen treatment toxicities. Elderly patients also more frequently harbor adverse cytogenetic features, which correlate with impaired DNA repair capacity and intrinsic chemoresistance.^46^ In this study, we demonstrated that lncRNA *AC021683.2* knockdown hindered Ara-C resistance in AML. Further analysis revealed that depletion of *AC021683.2* disrupts HSP90AA1-BCLAF1 interaction, promoting BCLAF1 ubiquitination and degradation, thereby enhancing Ara-C-induced apoptosis in resistant cells. Moreover, RAD50 mediates the role of *AC021683.2* and BCLAF1 in Ara-C-resistant AML cells. These findings establish the *AC021683.2*/BCLAF1/RAD50 axis as a pivotal therapeutic vulnerability in chemoresistant AML. Targeting this axis could boost leukemic cell sensitivity to lower Ara-C doses, reducing HiDAC needs—especially in elderly patients. By destabilizing BCLAF1 and blocking RAD50-mediated DNA repair, these strategies overcome chemoresistance while reducing toxicities—critical for vulnerable AML patients. This approach shows promise for treating relapsed/refractory AML by targeting DDR-mediated resistance and reducing chemotherapy side effects. Our study reveals a key Ara-C resistance mechanism and supports precision-guided low-dose chemotherapy, offering a strategy to improve AML outcomes.

### Limitations of the study

A primary limitation of this study is the small patient sample size (*n* = 10) in whole-transcriptome sequencing, which constrains statistical power and compromises the generalizability of the findings. Although RT-qPCR analysis of 18 Ara-C resistant/sensitive AML patients sample confirmed *AC021683.2* upregulation in resistant cases, which correlated with poor prognosis, the limited sequencing cohort may introduce potential sampling bias. This constraint limits comprehensive reflection of gene expression heterogeneity across the AML population. While current results show promise, validating *AC021683.2*’s biological significance requires testing in larger, genetically diverse clinical cohorts. Such efforts are crucial for minimizing false-negative results arising from small sample sizes. Additionally, integrating multi-omics data (genomic, transcriptomic, and epigenomic) and long-term clinical follow-up will enable precise identification of regulatory nodes in *AC021683.2*-mediated chemoresistance pathways. This holistic approach is essential for understanding the complex regulatory networks underlying chemoresistance in AML.

## Resource availability

### Lead contact

Further information and requests for resources and reagents should be directed to and will be fulfilled by the lead contact, Dr. Xiao-Ming Dong (xiaomingdong@snnu.edu.cn).

### Materials availability

This study did not generate any unique reagents.

### Data and code availability

• All RNA-seq raw data can be accessed in the SRA public database (SRA: PRJNA1111006).
• This paper does not report original code.
• Any additional information for reanalyzing the data in this study is available from the lead contact upon request.

## Acknowledgments

This work was funded by the 10.13039/501100001809National Natural Science Foundation of China (82472665, 82100218), Fundamental Research Funds for the Central Universities (GK202201004, GK202505003), the 10.13039/501100007128Natural Science Foundation of Shaanxi Province (2023-JC-YB-716), Excellent Graduate Training Program of 10.13039/501100004303Shaanxi Normal University (LHRCTS23091, LHRCCX23189). We thank Zhaoqiang Qian, Qiangqiang Wei, and Lifang Zheng from the Laboratory Animal Center of 10.13039/501100004303Shaanxi Normal University for their support and assistance in animal feeding, management and experiment.

## Author contributions

X.-M.D., P.G., and H.-L.T. conceived the project, L.L., X.-N.A., Y.-T.R., X.L., J.-L.H., J.-F.B., Y.-R.G., and X.L. performed the experiments. L.L., X.-M.D., and P.G. performed data analysis. X.-M.D., P.G., H.-L.T., and L.L. wrote the manuscript.

## Declaration of interests

The authors declare no competing interests.

## STAR★Methods

### Key resources table

**Table:** 

REAGENT or RESOURCE	SOURCE	IDENTIFIER
**Antibodies**		

BCLAF1 Polyclonal antibody	Proteintech Group	Cat# 26809-1-AP; RRID: AB_2880642
HSP90 Polyclonal antibody	Proteintech Group	Cat# 13171-1-AP; RRID: AB_2120924
RAD50 Polyclonal antibod	Proteintech Group	Cat# 29390-1-AP; RRID: AB_2918289
Monoclonal ANTI-FLAGⓇ M2 antibody	Sigma-Aldrich	Cat# F3165; RRID: AB_259529
Ubiquitin Rabbit mAb (A19686)	ABclonal	Cat# A19686; RRID: AB_2862735
GAPDH Monoclonal antibody	Proteintech Group	Cat# 60004; RRID: AB_2919223
Goat anti-Mouse IgG (H+L) HRP Secondary Antibody	Invitrogen	Cat# 31430; RRID: AB_228307
Goat anti-Rabbit IgG (H+L) HRP Secondary Antibody	Invitrogen	Cat# 31460; RRID: AB_228341
Anti-Ki67 Rabbit pAb	Servicebio	Cat# GB111499; RRID: AB_2927572
Mouse Control IgG	ABclonal	Cat# AC011; RRID: AB_2770414

**Biological samples**		

bone marrow samples	the Institute of Hematology, XiJing Hospital	N/A

**Chemicals, peptides, and recombinant proteins**		

Protease Inhibitor Cocktail (EDTA-Free, 100× in DMSO)	Targetmol	Cat# C0001
Puromycin	Yuanye Bio-Technology Co	Cat# P9620
Blasticidin S	Solarbio Life Science	Cat# B9300
RPMI 1640 medium	Thermo Fisher Scientific	Cat# 31800022
Iscove's Modified Dulbecco's Medium	Thermo Fisher Scientific	Cat# 12200036
High-glucose Dulbecco’s Modified Eagle Medium	Thermo Fisher Scientific	Cat# 12800017
Fetal Bovine Serum (Superfine), Uruguay	MedChemExpress	Cat# HY-T1000
Penicillin-Streptomycin Liquid	Solarbio Life Science	Cat# P1400
DAPI	Servicebio	Cat# G1012
Cytarabine	Solarbio Life Science	Cat# IC0630
MG-132	Targetmol	Cat# T2154
PVDF membranes	Pall Life Sciences	Cat# BSP0161
Polybrene	Sigma	Cat# TR1003
KCl	Yuanye Bio-Technology Co	Cat# R23154-500ml
Tris-HCl	Yuanye Bio-Technology Co	Cat# R21104-500ml
NaCl	Yuanye Bio-Technology Co	Cat# R21092-500ml
NP-40	Solarbio Life Science	Cat# N8030
0.5M EDTA (pH 8.0)	Solarbio Life Science	Cat# E1170

**Critical commercial assays**		

TUNEL Assay Kit	Servicebio	Cat# G1504
cDNA using the ABScript II cDNA First Strand Synthesis Kit	ABclonal	Cat# RK20400
Cell Counting Kit-8	TargetMol	Cat# C0005
SYBR Green qPCR Master Mix (No ROX)	TargetMol	Cat# C0006
Protein A/G Agarose Resin	YeasenBiotech	Cat# 36403ES
Dynabeads MyOne Streptavidin C1	Thermo Fisher Scientific	Cat# 65001
The Pierce BCA Protein Assay Kit	Thermo Fisher Scientific,	Cat# 23227
Clarity Western ECL Substrate	BIO-RAD	Cat# 1705061
APC-Annexin V/PI apoptosis kit	BIOSCIENCE	Cat# A6030L
T4 DNA Ligase	ABclonal	Cat# RK21501
Lipofectamine^2000^ transfection reagent	Thermo Fisher Scientific	Cat# 11668030
2 × Phanta Max Master Mix	Vazyme	Cat# P515-01
Trizol reagent	Takara	Cat# 9109

**Deposited data**		
RNA-Seq Raw Data	SRA	PRJNA1111006

**Experimental models: Cell lines**		

HEK293T	ATCC	Cat# CRL-1573; RRID:CVCL_0045
THP-1	ATCC	Cat# TIB-202
HL60	ATCC	Cat# CCL-240
HL60-ADR	Institute of Hematology of Tianjin	N/A

**Oligonucleotides**		

Primers for all, see Table S2	This paper	N/A

**Recombinant DNA**		

pLV3-CMV-MCS-3×FLAG-Blast	MIAOLING PLASMID	Cat# P51673
pLKO.1-TRC	Addgene	Cat# 10879; RRID:Addgene_10879
pCMV-VSV-G	Addgene	Cat# 8454; RRID:Addgene_8454
pMDLg/pRRE	Addgene	Cat# 12251; RRID:Addgene_12251
pRSV-Rev	Addgene	Cat# 12253; RRID:Addgene_12253

**Software and algorithms**		

GraphPad Software 8.0	GraphPad	https://www.graphpad.com
FlowJo (version 10.8.1)	BD Biosciences	https://www.flowjo.com/solutions/flowjo/downloads

### Experimental model and study participant details

#### Materials and methods

Diagnostic bone marrow samples from 28 patients with *de novo* AML were obtained from the Institute of Hematology, Xijing Hospital (Shaanxi, China)(No. KY20243348-1). Among them, 10 samples were used for whole-transcriptome sequencing (patient characteristics are summarized in Table 1), and 18 samples were used for gene expression validation (patient characteristics are summarized in Table S2). Bone marrow samples were selected from cases with sufficient cell numbers. Mononuclear cells were purified by standard Ficoll-Hypaque density centrifugation (GE Healthcare, Logan, UT, USA). Informed consent was provided according to the Declaration of Helsinki. All investigations were approved by the local ethics committee from Shaanxi normal university (Shaanxi, China).

#### Cell culture

HEK293T, THP-1 and HL60 cells were obtained from American Type Culture Collection (ATCC). HL60-ADR cells were obtained from the Institute of Hematology of Tianjin, Chinese Academy of Medical Sciences. HL60-ADR and THP-1 cells were cultured in RPMI 1640 medium. HL60 cells were maintained in Iscove's Modified Dulbecco's Medium. HEK293T cells were cultured in High-glucose Dulbecco’s Modified Eagle Medium. All media were supplemented with 10% FBS and 1% penicillin/streptomycin.

#### Xenograft model

All animal experiments on xenograft models were approved by the Shaanxi Normal University Ethics Committee (Approval No. 2024-076). We purchased 16 female BALB/c-nude mice(4-week-old)from Huafukang Bioscience Co (Beijing,China). The mice were randomly divided into groups after 7 days of adaptive feeding. 5×10^6^ THP-1 cells stably expressing sh*AC021683.2* or Control shRNA were resuspended in 150 uL PBS and injected into the flank of nude mice. The mice were divided into 4 groups:Control, sh*AC02683.2*, Control + Ara-C, sh*AC021683.2* + Ara-C (n=4 per group). As soon as the tumors became palpable, mice were injected intraperitoneally with Arc-C or vehicle (30 mg/kg/day). We measured the size of the xenograft tumor every three days and the tumor volume was calculated as follows:Volume(mm^3^)= (length × width^2^)/2. After 14 days of treatment, mice were sacrificed at the end of the experiment and xenograft tumors were harvested for subsequent analysis.

For mouse survival analysis experiment, 28 female BALB/c-nude mice (4 weeks) were injected with 2 × 10^6^ THP-1 cells stably expressing sh*AC021683.2* or Control shRNA via tail vein, respectively. The mice were treated with Ara-C or vehicle (50 mg/kg) for 10 days after THP-1 cells injection in the Control, sh*AC021683.2*, Control + Ara-C, sh*AC021683.2* + Ara-C (n=7 per group) groups. Survival days were monitored in each group and Kaplan-Meier survival plots were used to show survival.

### Method details

#### Whole transcriptome sequencing

Mononuclear cells from diagnostic bone marrow samples from patients with AML were collected and RNA extracted. RNA integrity and concentration were measured using the RNA Nano 6000 assay kit of the bioanalyzer 2100 system. Whole transcriptome sequencing was applied to the RNA samples with use of on an Illumina Novaseq 6000 platform in a 150-base double-end modeRNA at the Beijing Novogene. FASTQC software was used to screen the quality of raw data and keep high quality clean data for downstream analysis. Mapping and assembly of clean reads for each sample is first mapped to the reference genome with software HISAT2. The read alignment result is transferred to the program Stringtie for text assembly. All transcripts were merged using the CUFFMERGE software. LncRNAs were then identified from the assembled transcripts following four steps: (1) Removal of lowly expressed transcripts with FPKM < 0.5; (2) removal of short transcripts < 200 bp and < 2 exons; (3) removal of the transcripts with proteincoding capability using CNCI, Pfam and CPC2 database; (4) removal of the transcripts mapped within the 1 kb flanking regions of an annotated gene using Cuffcompare. lncRNAs were named following rules of HGNC. The characteristics of lncRNA was compared with known lncRNA and mRNA. Quantitative and differential expression analysis of transcripts and genes was performed by using StringTie software and Reads Per Kilobase of transcript per Million mapped reads (RPKM) was obtained. Cuffdiff or edgeR was used for differential expression analysis. The resulting *P*-values were adjusted using the Benjamini and Hochberg’s approach for controlling the false discovery rate. Genes with |log2 (Fold Change)| > 2 & padj < 0.05 were assigned as differentially expressed. Heat map function and volcano map were used to visualize differentially expressed gene clusters.

#### Antibodies and reagents

BCLAF1 (Cat# 26809-1-AP; Proteintech Group, Inc., Rosenont, IL, USA), Hsp90AA1(Cat# 13171-1-AP; Proteintech Group, Inc., Rosenont, IL, USA), RAD50(Cat# 29390-1-AP; Proteintech Group, Inc., Rosenont, IL, USA), Flag-tag (Cat# F3165; Sigma-Aldrich, Darmstadt, Germany), Ub (Cat# A19686; ABclonal, Wuhan, China), GAPDH (Cat# 60004; Proteintech Group, Inc. , Rosenont, IL, USA), Goat anti-Mouse IgG (H+L) HRP Secondary Antibody (Cat# 31430; Invitrogen, Carlsbad, CA, USA), Goat anti-Rabbit IgG (H+L) HRP Secondary Antibody (Cat# 31460; Invitrogen, Carlsbad, CA, USA), Ki-67 Antibody (Cat# GB111499; Servicebio, Wuhan, China), DAPI (Cat# G1012; Servicebio, Wuhan, China), TUNEL Assay Kit(Cat# G1504; Servicebio, Wuhan, China). Ara-C was purchased from Solarbio Life Science, MG132 was purchased from Targetmol.

#### Quantitative real-time PCR

Total RNA was extracted from mononuclear cells of AML patients and cell lines using Trizol reagent (Cat# 9109; Takara, Tokyo, Japan). 2 μg of RNA was reverse transcribed into cDNA using the ABScript II cDNA First Strand Synthesis Kit (Cat# RK20400; ABclonal, Wuhan, China). SYBR-Green qPCR analysis was performed using SYBR Green qPCR mix (Cat# C0006; TargetMol, Shanghai, China) in accordance with the manufacturer's protocol. *β-actin* was used as an internal control. Sequences of all primers used in this study are listed in Table S3.

#### Cell proliferation assay and IC50 determinations

For cell proliferation assay, cells were counted and seeded into 96-well plates at a density of 1 × 10^3^ cells/well. Then cell proliferation was measured at 0 h, 24 h, 48 h, 72 h using the CCK-8 Assay Kit (Cat# C0005; TargetMol, Shanghai, China). For IC50 determination,All cells in logarithmic growth stage were distributed at 1×10^4^ cells/well into 96-well plates under treatment with Ara-C at different concentrations(0 μM, 0.1 μM, 0.2 μM, 0.4 μM, 0.8 μM, 1.6 μM, 3.2 μM, 6.4 μM, 12.8 μM)for 48 h (4 replicates per group) . After addition of fresh mediun containing 10% CCK-8 for 4 h, the absorbance at 450nm for each well were measured by the Multifunctional Microplate Reader (BioTek, USA), and then the curve between Ara-C concentration and cell survival was plotted, the IC50 values were calculated by Graphpad Prism 10.1.1 software.

#### Apoptosis assay

AML cell lines were cultured in medium with/without Ara-C (5 μM) for 48 h. Then cells were centrifuged at 1500 rpm/min for 5 min, and washed with cold PBS twice to remove residues in the medium and cell culture. The cells were collected and resuspended in 500 μL binding buffer and incubated with 5 μL Annexin V-APC and 5 μl PI (Cat# A6030L; BIOSCIENCE, Shanghai, China) staining solution for 15 min at room temperature in the dark. The samples were then immediately analyzed by Flow cytometry. All data were analyzed using FlowJo software.

#### Western blot

Western blot was performed as described previously.^47^ Briefly, cells were harvested and lysed in ice-cold cell lysis buffer (20 mM Tris-HCl, pH7.4; 0.5 M NaCl; 1% NP-40; 5 mM EDTA, pH 8.0; Protease Inhibitor; 1mM DTT), followed by brief sonication and then centrifugation to remove insoluble material. Protein concentration was detected by BCA Protein Assay Kit (Cat# 23227; Thermo Fisher Scientific, Waltham, MA, USA). Protein samples were separated on 10% SDS-PAGE and transferred to PVDF membranes (Cat# BSP0161; Pall Life Sciences, Port Washington, New York, USA). After blocking with 5% nonfat milk in TBST, the membranes were incubated with specific primary antibodies overnight at 4°C. After washing off the primary antibodies, the membranes were then incubated with secondary antibodies for 1 h at room temperature. Immunoreactive bands on blots were visualized using ECL Western Blotting Substrate (Cat# 1705061; Bioad, CA, USA).

#### Plasmids, lentivirus production and Infection

For gene overexpression experiments, full-length human *BCLAF1*, and *RAD50* amplified by PCR were cloned into the pLV3-CMV-MCS-3×FLAG-Blast vector, respectively. For knockdown experiments, *AC021683.2*, *BCLAF1* and *RAD50* targeting shRNA designed using shRNA sequence designer software and the oligonucleotide were commercially synthesized and then cloned to the pLKO.1-TRC vector, respectively. To minimize the off-target effects of lncRNAs, we designed two shRNAs. One shRNA is specifically designed to target the 3' untranslated region (3'UTR), while the other targets the first exon of the selected lncRNA, *AC021683.2*. The 3'UTR region is a highly conserved sequence, and this conservation contributes to reducing the off-target effects of the lncRNAs. Briefly, HEK293T cells were co-transfected with 1.5μg shRNA/overexpression vector, 0.5 μg pLP/VSVG (envelope plasmid), 0.5 μg pMDLg/pRRE (package plasmid) and 0.5 μg pRSV-Rev (package plasmid) using Lipofectamine^2000^ transfection reagent (Cat# 11668030; Thermo Fisher Scientific, Waltham, MA, USA) for 6h.The viral supernatants from day 2 to day 4 was harvested and centrifuged for 5 min(2000 rpm/min), filtered through 0.45 μm filter and stored at -80 °C. AML cell lines were infected overnight with viral supernatant in the presence of 4 μg/mL polybrene (Cat# TR1003; Sigma, St. Louis, MO, USA) and cultured in fresh medium for another 24 h. Cells were selected with complete medium containing 1 μg Puromycin (Cat# P9620; Yuanye Bio-Technology Co, Shanghai, China).When uninfected control AML cells were totally dead, the target cells were placed in the normal culture medium for further experiments. The primer sequences used for amplification and shRNA sequences targeting genes are listed in Table S3.

#### Hematoxylin and eosin (H&E)

The sections were removed from paraffin blocks and placed in xylene for 5-10 min, then washed with anhydrous ethanol, 95%, 85% and 70% ethanol and ddH_2_O. Each section was washed 3 times with PBS. Each section was stained with hematoxylin for 10 minutes and then washed. The slides were then stained with eosin solution, washed with gradient ethanol and cleared of xylene. The slides were mounted with a neutral resin. Finally, the microscope was used to observe and film the sections.

#### Immunohistochemistry (IHC) and TUNEL analyses

Sections were soaked in xylene for 10 min and washed with an ethanol gradient (100-70%) and incubated with antigen retrieval solution and 3% H_2_O_2_ for 10 min. Sections were washed with ddH_2_O and incubated with Ki-67 antibody (1:800) at 4 °C overnight. Sections were washed with PBS and added secondary antibodies for 60 min at room temperature and incubated in DAB for 5-10 min, and stained with hematoxylin. The slices were then washed with ddH_2_O and gradient alcohol and cleared in xylene. Finally, the slices were sealed with a neutral resin and viewed with Fluorescence microscope.

For TUNEL assay, sections were placed in xylene and washed with an ethanol gradient (100-70%) and ddH_2_O. After the section is slightly dried, draw a circle around the tissue with a histochemical pen, add protease K working liquid in the circle to cover the tissue, and incubate for 20min. The slide was placed in PBS and washed by shaking on the decolorizing shaker for 3 times, 5min each time. After the section is slightly dried, the tissue is covered with the broken film working liquid in the circle, incubated at room temperature for 20min, and the slide is placed in PBS and washed by shaking on the decolouring table for 3 times, for 5min each time. The sections were labeled with TUNEL reaction mixture to detect apoptotic cells. After the TUNEL reaction, sections were washed three times with PBS and incubated in DAPI solution to label nuclear DNA. Then the slices are sealed and viewed with Fluorescence microscope.

#### RNA-pull down assay

1×107 HL60-ADR cells were collected and lysed in ice-cold cell lysis buffer (20 mM Tris HCl, pH 7.5; 150 mM NaCl; 1 mM EDTA; 0.5% NP-40)with protease inhibitor and RNase inhibitor for 10 min. Cell lysate was precleared with Magnetic beads (Cat# 65001; Thermo Fisher Scientific, Waltham, MA, USA). Buffer A (150 mM kCl; 25 mM Tris HCl, pH 7.5; 5 mM EDTA; 0.5% NP-40; Protease Inhibitor and RNase Inhibitor) was added to containing biotinylated DNA each sample in a 1:1 ratio and incubated for 4 h with rotation at 4 °C. DNA probes of AC021683.2 were designed using PaintSHOP. Probes with 3' biotinTEG modification were purchased from Beijing Tsingke Biotech (probe sequences are listed in Table S3). Magnetic beads were then added to the lysate and incubated at room temperature for an additional 45 min with rotation. Beads-probe-RNA-protein complexes were collected using DynaMag-15 magnetic strip and washed 5 times with Buffer A. 10% beads were used for RNA isolation with TRIzol and 90% beads were used for protein elution and downstream analysis,The mass spectrometry results are presented in Table S4.

#### Immunoprecipitation

Cells were harvested and lysed in ice-cold cell lysis buffer (20 mM Tris-HCl, pH7.4; 0.5 M NaCl; 1% NP-40; 5 mM EDTA, pH 8.0; Protease Inhibitor; 1 mM DTT). After brief sonication, the lysates were centrifuged at 12000g for 15 min at 4°C. Supernatants were incubated with anti-Hsp90AA1 antibody and protein A/G (Cat# 36403ES; YeasenBiotech, Shanghai, China) beads overnight at 4°C. After immunoprecipitation, the beads were washed three times with the lysis buffer, and the precipitates were analyzed by western blot. For detecting the endogenous interaction, whole HL60-ADR cell lysis was immunoprecipitated with anti-Hsp90AA1 antibody overnight at 4°C. Normal IgG (Cat# AC011; ABclonal, Wuhan, China) was used as a negative control.

#### Ubiquitination of BCLAF1

HL60-ADR cells were transfected with shcontrol, sh*AC021683.2*, and cotransfected with BCLAF1-PLV3-Flag plasmid in the presence or absence of 5μM Ara-C. The cells were collected and lysed in lysis buffer, respectively. Then, the ubiquitination of BCLAF1 was analyzed by immunoprecipitation with anti-Flag antibody and immunoblotted with anti-Ub (Cat# A19686; ABclonal, Wuhan, China) antibody or anti-Flag antibody.

### Quantification and statistical analysis

All statistical analyses were performed using GraphPad Prism 10.1.1. All data are presented as mean ± SD. All comparisons were tested using unpaired two-tailed Student's *t* test or one-way ANOVA as described in the figure legends. Mouse survival was analyzed by Kaplan-Meier analysis. Image J was used to calculate the mean density of Ki-67 positive signals in immunohistochemical sections. *P* < 0.05 was considered statistically significant. ∗ signifies *P* < 0.05, ∗∗ *P* < 0.01, ∗∗∗ *P* < 0.001. All experiments were repeated at least three times.

## Supplemental information

Table S1. Differentially expressed genes between IA-sensitive and IA-resistant samples, related to Figure 1

Table S2. Clinical characteristics of patient cohorts, related to Figure 1

Table S3. The sequence of primers

Table S4. Mass spectrometry analysis results, related to Figure 4
